# A Genetic Biomarker of Oxidative Stress, the Paraoxonase-1 Q192R Gene Variant, Associates with Cardiomyopathy in CKD: A Longitudinal Study

**DOI:** 10.1155/2016/1507270

**Published:** 2016-05-30

**Authors:** E. Dounousi, I. Bouba, B. Spoto, K. Pappas, G. Tripepi, I. Georgiou, A. Tselepis, M. Elisaf, D. Tsakiris, C. Zoccali, K. Siamopoulos

**Affiliations:** ^1^Department of Nephrology, University Hospital of Ioannina, 45110 Ioannina, Greece; ^2^Laboratory of Clinical Genetics and Human Reproduction, Medical School University of Ioannina, 45110 Ioannina, Greece; ^3^CNR-IFC, Clinical Epidemiology and Physiopathology of Renal Diseases and Hypertension, 89124 Reggio Calabria, Italy; ^4^Department of Cardiology, University Hospital of Ioannina, 45110 Ioannina, Greece; ^5^Laboratory of Biochemistry, Department of Chemistry, University of Ioannina, 45110 Ioannina, Greece; ^6^Department of Internal Medicine, University Hospital of Ioannina, 45110 Ioannina, Greece; ^7^Department of Nephrology, General Hospital “Papageorgiou” of Thessaloniki, 564 29 Thessaloniki, Greece

## Abstract

*Background*. Oxidative stress is a hallmark of CKD and this alteration is strongly implicated in LV hypertrophy and in LV dysfunction.* Methods and Patients*. We resorted to the strongest genetic biomarker of paraoxonase-1 (PON1) activity, the Q192R variant in the PON1 gene, to unbiasedly assess (Mendelian randomization) the cross-sectional and longitudinal association of this gene-variant with LV mass and function in 206 CKD patients with a 3-year follow-up.* Results*. The R allele of Q192R polymorphism associated with oxidative stress as assessed by plasma 8-*iso*PGF_2*α*_ (*P* = 0.03) and was dose-dependently related in a direct fashion to LVMI (QQ: 131.4 ± 42.6 g/m^2^; RQ: 147.7 ± 51.1 g/m^2^; RR: 167.3 ± 41.9 g/m^2^; *P* = 0.001) and in an inverse fashion to systolic function (LV Ejection Fraction) (QQ: 79 ± 12%; RQ: 69 ± 9%; RR: 65 ± 10% *P* = 0.002). On longitudinal observation, this gene variant associated with the evolution of the same echocardiographic indicators [LVMI: 13.40 g/m^2^ per risk allele, *P* = 0.005; LVEF: −2.96% per risk allele, *P* = 0.001]. Multivariate analyses did not modify these associations.* Conclusion*. In CKD patients, the R allele of the Q192R variant in the PON1 gene is dose-dependently related to the severity of LVH and LV dysfunction and associates with the longitudinal evolution of these cardiac alterations. These results are compatible with the hypothesis that oxidative stress is implicated in cardiomyopathy in CKD patients.

## 1. Introduction

Left ventricular hypertrophy (LVH) and LV systolic dysfunction as quantified by Ejection Fraction (LVEF) are solidly established surrogate markers of cardiovascular disease [1–3] and these measurements have been extensively applied as end-points in major clinical trials in cardiology and in nephrology [4–6]. In patients with chronic kidney disease (CKD), these biomarkers are held as strong risk factors for cardiovascular disease at both the predialysis [7, 8] and dialysis [9, 10] stages. These alterations are multifactorial in nature and share a variety of risk factors including afterload related factors (hypertension and arterial stiffness), volume overload, CKD-mineral bone disorders (CKD-MBD) like hyperphosphatemia, hyperparathyroidism, reduced vitamin D receptor activation and high Fibroblast Growth Factor 23 (FGF23), sympathetic overactivity, nitric oxide (NO) inhibition by the accumulation of endogenous inhibitors of NO synthase, inflammation, and oxidative stress [11]. Among these risk factors, oxidative stress appears of particular relevance because this pathway is a potential common route whereby the same factors, including CKD-MBD disorders [12, 13], pressure overload, and NO inhibition [14], may influence myocardial growth, fibrosis, and ultimately LV function. However, evidence that oxidative stress may impinge upon LV mass and function in CKD is mainly limited to animal experiments focusing on CKD-MBD-related risk factors [12, 13].

Paraoxonase-1 (PON1), a major antioxidant, is regarded as a cardiovascular protective factor and it is well documented that the activity of this enzyme is reduced in patients with cardiovascular diseases [15, 16]. Paraoxonase levels are genetically regulated. The Q192R polymorphism in the PON1 gene is held as a main genetic biomarker of oxidant status. Detailed biochemical studies documented that the Q allele codifying for the Q isoenzyme underlies a higher antioxidant effect than the allele codifying for the R isoenzyme [17] and that protection from oxidation of low density lipoprotein is maximal in homozygous QQ subjects and minimal in RR subjects [18]. A meta-analysis documented that the Q192R polymorphism is the sole genetic variant in the PON1 gene to show a highly significant, albeit small (7% per R allele), risk for coronary heart disease [19] and another independent meta-analysis estimated a 10% risk excess per R allele for the same outcome [20]. The R allele associates with low HDL levels [21, 22] as well as with albuminuria and reduced eGFR in women [23]. We hypothesized that, in addition to coronary heart disease and perhaps renal disease, the prooxidant state generated by low paraoxonase activity may also favor LVH and LV systolic dysfunction by direct and indirect mechanisms. A small study in hemodialysis patients reported that LV mass is inversely related to paraoxonase activity [24]. On the other hand, in a large study in patients with systolic dysfunction, low activity of this enzyme more than doubled the risk of cardiac events over a three-year follow-up [25]. However, these observations are purely observational and open to bias and confounding because paraoxonase activity is influenced by several environmental factors including diet, smoking, and other factors [15] and therefore represent only weak evidence that oxidative stress by reduced paraoxonase activity is causally implicated in LV disorders in CKD.

Since genes are transmitted randomly at gamete formation, genetic polymorphisms represent a valid approach to exploring causality in human disease [26]. For this reason, we resorted to the strongest genetic biomarker of paraoxonase activity discovered so far, the missense mutation in PON1 gene coding for the Q192R variant (rs662; i.e., A and G alleles coding for glutamine (Q) and arginine (R), resp.) [19, 20] to unbiasedly assess the association between paraoxonase activity with LV mass and function and the relationship of the same gene variant with the temporal evolution of LVH and systolic dysfunction in a longitudinal study in a cohort of 206 patients with CKD.

## 2. Patients and Methods

### 2.1. Study Population

Two hundred twenty-nine consecutive, incident adult CKD patients from the Renal Outpatient Clinics of two hospitals were included in this longitudinal study. Patients were genetically homogenous all being of Caucasian descent and from the same geographical area, northwest Greece. Prior to the study, inclusion patients were followed up for three months to confirm the diagnosis of CKD. None had major cardiovascular events including stroke, peripheral vascular disease, angina, and myocardial infarction during the three months preceding enrollment. NYHA stage IV, acute intercurrent infection, or acute inflammatory processes at recruitment and history of malignancy during the five years preceding the study were the sole exclusion criteria.

The study was approved by the Ethical Committees of the two hospitals and patients participated in the study after providing written informed consent.

### 2.2. Echocardiography

Echocardiography was performed annually for up to 3 years by a single experienced cardiologist from each hospital, who was blinded to the clinical and biochemical data. Echocardiographic parameters were measured by standard methods described in detail previously [27]. Left ventricular systolic function was estimated by measuring the Ejection Fraction (LVEF, %) [28].

### 2.3. Oxidative Stress Assessment

Plasma 8-isoprostanes (8-*iso*PGF_2*α*_) levels were used as an indicator of oxidative stress [29] and were measured by a competitive enzyme immunoassay (ELISA) (Cayman Chemicals, Ann Arbor, MI, USA).

### 2.4. Genotyping of Q192R Polymorphism

Genomic DNA was extracted from peripheral blood leukocytes by using NucleoSpin Blood QuickPure kit (Macherey-Nagel GmbH & Co. KG, Düren, Germany). Patients were genotyped for a common single nucleotide polymorphism (C672T) in PON1 gene coding for the Q192R protein variant. This polymorphism, described under identification number rs662, was studied by the predesigned TaqMan SNP Genotyping Assay C_2548962_20 provided by Applied Biosystems (Applied Biosystems, Foster City, CA, USA). Allelic discrimination was performed by the Rotor-Gene 3000 system (Corbett Research, Mortlake, Australia).

### 2.5. Statistical Analysis

The relationship between Q192R polymorphism and LVMI and LVEF was evaluated in two steps: (1) at baseline and (2) prospectively by using repeated measurements of the outcomes.

At baseline, unadjusted and adjusted linear regression models were used to test the association between the Q192R variant (codominant model) and the study outcomes (LVMI and LVEF). In the adjusted models, we included variables which were related to the exposure (i.e., Q192R polymorphism) with *P* ≤ 0.10 (see Table 1) (i.e., HDL and eGFR) at univariate analysis.

The longitudinal association between Q192R polymorphism and the evolution of LVMI and LVEF over time was investigated by linear mixed models (LMM). In these analyses, the intrasubjects correlation was modeled by fitting different variance-covariance structures and the structure associated with the lowest Akaike information criterion was adopted. In LMM, we adjusted for the same set of variables considered in the linear regression model.

## 3. Results

The original cohort was formed by 229 patients. Twenty-three patients with inadequate acoustic window for echocardiographic examination, severe valve heart disease, and unwillingness to undergo echocardiography at baseline were excluded from the study, leaving 206 patients for analysis. These patients (mean age of 65) were distributed among the five CKD stages as follows: stage G1, 11%; stage G2, 29%; stage G3, 36%; stage G4, 22%; stage G5, 2%. One hundred threepatients were males (50%), 64 had type 2 diabetes (31%), 33 were active smokers (16%), and 55 had cardiovascular comorbidities (27%) (Table 1). The vast majority (91%) was receiving antihypertensive treatment and 74 patients (36%) were on statin therapy. The mean eGFR was 54 ± 30 mg/mL/1.73 m^2^ and the median proteinuria was 270 mg/24 hours (IQR, 130–1080 mg/24 hours). The median plasma 8-*iso*PGF_2*α*_ level was 106 pg/mL (IQR, 90–134 pg/mL).

The genotype distribution of the Q192R (rs662; A/G) polymorphism [QQ, *n* = 110 (53.4%); QR, *n* = 83 (40.3%); RR = 13 (6.3%)] did not deviate from the Hardy-Weinberg equilibrium (*x* ^2^ = 0.26, *P* = 0.61). Table 1 summarizes the characteristics of the patients stratified according to Q192R genotypes at enrollment. Because transmission gene variants are a random phenomenon, patients among the three genotypes did not significantly differ as for demographic and clinical characteristics including age, sex, BMI, diabetes, smoking, hypertension, cardiovascular comorbidities, and medications (Table 1). Like in previous studies [22, 23], the R allele of the Q192R polymorphism tended to associate with HDL cholesterol and the eGFR, but these associations failed to achieve statistical significance (*P* = 0.09 for both associations).

Plasma 8-*iso*PGF_2*α*_ levels, an established marker of the endogenous lipid peroxidation, in patients homozygous for the R allele (RR: 136 pg/mL IQR 112–165) were higher (*P* = 0.03 by ANOVA) than in patients without (QQ: 109 pg/mL; 103–134) or with just one R allele (QR: 102 pg/mL; 84–128 pg/mL).

## 4. Baseline Associations of Q192R Polymorphism with LVMI and EF

**Figure 1:**
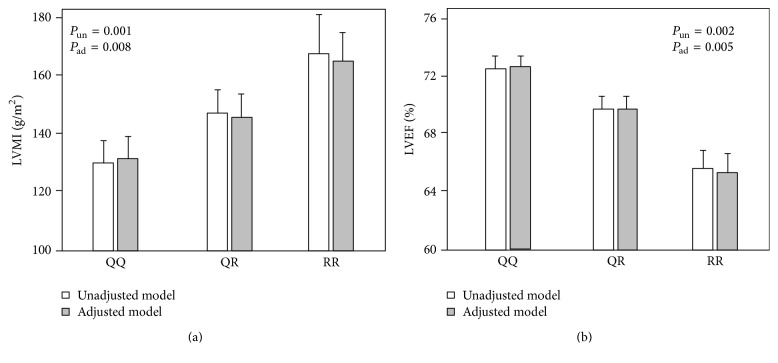
Relationship between the Q192R polymorphism and LVMI (a) and LVEF (b).

There was a dose-response relationship between the number of R alleles and LVMI [Figure 1: unadjusted model, white bars], so that patients with the RR192 genotype had on average the highest LVMI (167.3 ± 41.9 g/m^2^), heterozygous RQ patients (147.7 ± 51.1 g/m^2^) the intermediate LVMI value, and homozygous QQ patients (131.4 ± 42.6 g/m^2^) the lowest LVMI (*P* = 0.001). Alterations in LVEF across Q192R genotypes mirrored those in LVMI and LVEF was on average lowest in homozygous RR patients (65 ± 10%), intermediate in heterozygous RQ patients (69 ± 9%), and highest in homozygous QQ patients (72 ± 9%) (*P* = 0.002) [Figure 1: unadjusted model, white bars].

To explore whether the relationship between the R allele and LV mass and function could be mediated by the effect of the same allele on HDL and eGFR, we performed a multiple regression analysis adjusting for these parameters (Table 2). In this analysis, the R allele remained a strong, independent correlate of LVMI and LVEF and the regression coefficients in the adjusted analyses (LVMI *b* = +17.06 g/m^2^ per risk allele and LVEF *b* = −3.12% per risk allele) reduced very modestly as compared to those registered in the unadjusted analysis (+13.3 g/m^2^ and −2.91%, resp.) [Table 2 and Figure 1; adjusted models, grey bars] indicating that the hypothetic action of this allele on LV mass and function was largely independent of the effects of the same allele on lipid metabolism and renal function.

## 5. Longitudinal Associations of Q192R Polymorphism and LVMI and LVEF

Of the 206 patients enrolled into the study, 7 patients died and could be studied only at baseline and the same was true for 12 patients who started dialysis and 20 patients who were lost to follow-up. All remaining patients had at least one follow-up echocardiographic study. Overall, a total of 500 LVMI and LVEF measurements were available for the longitudinal, mixed linear modeling analysis. In both the unadjusted and adjusted longitudinal analyses, the Q192R polymorphism coherently predicted the evolution of LVMI and LVEF over time (Table 3). In detail, in the unadjusted analysis for one risk allele increase (R allele), the corresponding increase of LVMI was 13.4 g/m^2^ over the average 2.4-year follow-up (*P* = 0.005) and the corresponding decrease of LVEF was −2.96% (*P* = 0.001). The multivariate analysis aimed at exploring if low HDL and eGFR could have been involved in mediating the effect of the R allele on LV mass and function, largely confirming that these coexisting effects of the same allele were immaterial to explain the strong relationship between the evolution of LVH and LV systolic dysfunction in the longitudinal study (Table 3).

## 6. Discussion

This study in a genetically homogenous group of patients with CKD of various severity shows that the R allele of Q192R variant in the PON1 gene is dose-dependently related to LV mass (in a direct fashion) and with systolic function (in an inverse fashion). Furthermore, on longitudinal observation, this gene variant associates with the evolution of the same echocardiographic indicators over follow-up. Overall, these results are in line with the hypothesis that oxidative stress triggered by this polymorphism plays a role in cardiomyopathy in CKD patients.

Experimental studies in animal models coherently demonstrated that reactive oxygen species stimulate myocardial growth, matrix remodeling, and myocardiocyte dysfunction [14]. Molecular signaling pathways linking oxidative stress to cardiac hypertrophy and remodeling include a variety of signaling kinases and transcription factors [31]. Among these pathways, endothelial NO synthase (eNOS) is a crucial signal because uncoupling of eNOS by oxidative stress activates myocardiocyte hypertrophy and metalloproteinases which in turn degrade extracellular matrix, thereby favoring ventricular dilation and dysfunction [32].

Oxidative stress is seen as a pervasive problem in patients with kidney failure [33]. High levels of reactive oxygen species reduce NO bioavailability and by direct and indirect mechanisms accelerate the progression of atherosclerosis in this population [34].

**Figure 2:**
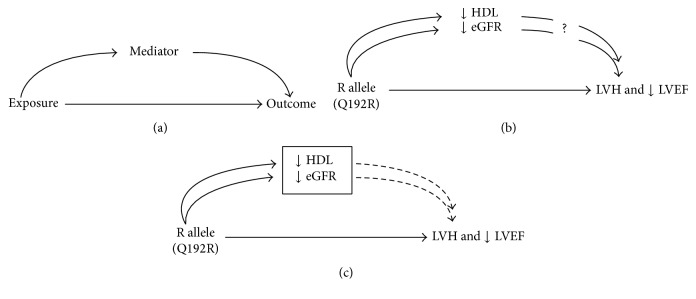
Directed acyclic graph [30] illustrating the relationship between the R allele of the Q192R polymorphism and ↓HDL, ↓eGFR, LVH, and ↓LVEF. (b) shows that the R allele associates (likely in a causal manner) with all outcomes and in theory it is possible that the effect of this allele on cardiac parameters is mediated in part or in full via ↓HDL and ↓eGFR. (c) Adjustment (box, which controls for HDL and eGFR) has minimal or no effect (Tables 2 and 3) on the link between the R allele and LVMI and LVEF suggesting that this link is a direct effect of this allele on the heart.

To explore the hypothesis that high levels of reactive oxygen species are implicated in LVH and LV dysfunction in CKD patients, we exploited a missense mutation in the PON1 gene coding for the Q192R variant which has been functionally associated with oxidative stress [15–17] and coronary heart disease [17, 20, 22, 35]. The frequency of the risk allele in our cohort did not deviate from Hardy-Weinberg equilibrium and did not differ from that in the general population of European ancestry in the HAPMAP (67% versus 74%). In line with our working hypothesis, we found a dose-dependent relationship between the risk allele (R) of the Q192R polymorphism and LVMI in CKD. This finding was paralleled by increasing degrees of LV systolic dysfunction in patients classified according to the number of copies of the same (R) risk allele. Due to Mendelian randomization [26], these associations were largely unconfounded by traditional and CKD specific risk factors (Table 1). Furthermore, additional analyses (multivariate analysis, Tables 2 and 3) aimed at exploring how much the effect of the R allele on the left ventricle could have been mediated via the action of the same allele on HDL levels [22] and/or on the eGFR [23] showed that these factors had minimal, if any, influence on the association of the same allele with LV mass and function. This observation, which is illustrated by a directed acyclic graph [30] (Figure 2), may suggest that effect of the R allele on the heart is a direct phenomenon rather than a phenomenon mediated via dyslipidemia or renal dysfunction.

Observations by Park in the CRIC study showed that the prevalence of LVH goes along with the severity of CKD while LVEF does not [7], suggesting that progressive myocardial hypertrophy may serve to compensate the effect of factors which might otherwise compromise systolic function. Due to the exclusion of patients with NYHA IV, the average baseline LVEF in our cohort was well within the normal range and like in Park et al.'s study [7] did not correlate with CKD stages. However, like LVMI, LVEF in our patients associated dose-dependently with the number of R alleles of the Q192R polymorphism, a variant associated with reduced antioxidant potential [17].

Longitudinal observations confirming the association between the genetic marker of oxidative stress (Q192R) and the evolution of LVH and LV systolic dysfunction over time add strength to the hypothesis that these links might underlie a causal effect. Longitudinal studies provide more robust evidence for causal claims, particularly in the setting of genetic epidemiology studies that is studies where random gene assortment at gamete formation generates an experimental setting mimicking the clinical trial [26]. Our findings based on two well-validated markers of cardiomyopathy (LVMI and LVEF) may be relevant for the high risk of cardiovascular complications of CKD patients. Reduced paraoxonase activity emerged as a powerful, independent predictor of death and incident cardiovascular events including myocardial infarction and stroke in a large cohort of CKD patients [36].

This study has limitations. Our CKD cohort was relatively small, particularly the number of patients that could be entered into in the longitudinal analyses, and was composed by patients of Caucasian descent enrolled in a restricted geographical area. More importantly, we did not replicate our findings in a second cohort. However, as alluded to before, the coherent association between the Q192R variant with LV mass and function at baseline and during longitudinal observation would support the contention that this association is robust and reliable. Nonetheless, the generalizability of our findings in the CKD population awaits confirmation in other, larger CKD cohorts and in other ethnicities. Furthermore, we did not measure paraoxonase activity, the intermediate mechanism whereby the gene variant in question increases oxidative stress. Yet, we confirmed the expected link between Q192R and oxidative stress as measured by 8-*iso*PGF_2*α*_ levels.

In conclusion, in CKD patients, the R allele of Q192R variant in the PON1 gene is dose-dependently related to the severity of LVH and LV dysfunction and associates with the longitudinal evolution of these cardiac alterations. These results are in line with the hypothesis that implicates oxidative stress in cardiomyopathy in CKD patients. Larger cohort studies and a clinical trial in CKD patients looking at clinical end-points like death and cardiovascular events are needed to support the hypothesis that oxidative stress is causally implicated in cardiomyopathy in this population.

## Figures and Tables

**Table 1:** Main demographic and clinical characteristic of the study population grouped according to Q192R polymorphism.

	Q192R polymorphism				
	Whole group (*n* = 206)	QQ (*n* = 110)	QR (*n* = 83)	RR (*n* = 13)	*P*
Age (years)	65 ± 12	64 ± 12	65 ± 13	67 ± 11	0.18
Male sex, *n* (%)	103 (50)	51 (46.4)	43 (51.8)	9 (69.2)	0.14
Diabetes, *n* (%)	64 (31)	36 (33)	26 (31)	2 (15)	0.34
Smokers, *n* (%)	33 (16)	20 (18.2)	12 (14.5)	1 (7.7)	0.28
Cardiovascular comorbidities, *n* (%)	55 (27)	28 (25)	23 (28)	4 (31)	0.63
BMI (kg/m^2^)	29.3 ± 5.3	29.3 ± 5.1	29.4 ± 5.6	28.3 ± 4.6	0.75
Systolic BP (mmHg)	141 ± 19	141 ± 18	139 ± 18	145 ± 25	0.82
Diastolic BP (mmHg)	81 ± 10	81 ± 11	81 ± 10	79 ± 13	0.81
On antihypertensive therapy, *n* (%)	188 (91)	100 (91)	75 (90)	13 (100)	0.41
On statin therapy, *n* (%)	74 (36)	41 (37)	29 (35)	4 (31)	0.61
Total cholesterol (mg/dL)	214 ± 45	215 ± 45	214 ± 45	208 ± 49	0.74
HDL (mg/dL)	52 ± 13	54 ± 14	51 ± 11	51 ± 12	0.09
LDL (mg/dL)	129 ± 36	129 ± 36	130 ± 37	123 ± 36	0.83
Triglycerides (mg/dL)	143 (101–193)	132 (98–194)	150 (108–193)	127 (112–222)	0.51
Glucose (mg/dL)	116 ± 48	115 ± 41	120 ± 58	110 ± 33	0.82
Haemoglobin (g/dL)	13.3 ± 1.6	13.3 ± 1.6	13.3 ± 1.7	13.0 ± 1.4	0.65
Albumin (g/dL)	4.2 ± 0.3	4.2 ± 0.3	4.2 ± 0.4	4.3 ± 0.3	0.87
Calcium (mg/dL)	9.4 ± 0.6	9.4 ± 0.6	9.4 ± 0.6	9.4 ± 0.6	0.99
Phosphate (mg/dL)	3.4 ± 0.7	3.4 ± 0.6	3.4 ± 0.7	3.6 ± 0.8	0.84
Parathormone (pg/mL)	72 (47–115)	71 (45–113)	73 (45–117)	103 (61–131)	0.27
CRP (mg/L)	2.0 (0.9–5.0)	2.0 (0.9–5.0)	2.0 (0.8–4.6)	1.5 (0.2–4.0)	0.39
eGFR MDRD_186_ (mL/min/1.73 m^2^)	54 ± 30	57 ± 30	52 ± 30	45 ± 25	0.09
Urine protein (mg/24 h)	270 (130–1080)	295 (127–1119)	273 (140–972)	195 (124–2091)	0.89

Data are expressed as mean ± SD, median and interquartile range, or percent frequency, as appropriate.

**Table:** (a) LVMI model

Variables (unit of increase)	Unadjusted model			Adjusted model		
	*b* (95% CI)	*β*	*P*	*b* (95% CI)	*β*	*P*
Q192R polymorphism (one risk allele increase)	17.06 (6.74 to 27.39)	0.22	0.001	13.30 (3.43 to 23.17)	0.17	0.008
HDL (1 mg/dL)				−0.45 (−0.93 to 0.03)	−0.12	0.07
eGFR MDRD_186_ (1 mL/min/1.73 m^2^)				−0.47 (−0.68 to −0.27)	−0.30	<0.001

**Table:** (b) LVEF model

Variables (unit of increase)	Unadjusted model			Adjusted model		
	*b* (95% CI)	*β*	*P*	*b* (95% CI)	*β*	*P*
Q192R polymorphism (one risk allele increase)	−3.12 (−5.12 to −1.11)	−0.21	0.002	−2.91 (−4.92 to −0.91)	−0.20	0.005
HDL (1 mg/dL)				0.13 (0.03 to 0.23)	0.18	0.01
eGFR MDRD_186_ (1 mL/min/1.73 m^2^)				−0.02 (−0.06 to 0.02)	−0.06	0.39

Data are expressed as unstandardized regression coefficient (*b*) and 95% confidence interval, standardized regression coefficient (*β*), and *P* value.

**Table:** (a) LVMI model

Variables (units of increase)	Model 1 (unadjusted)		Model 2	
	*b*	*P* value	*b*	*P* value
PON1 (Q192R)	13.40 (4.17 to 22.62)	0.005	9.24 (0.51 to 17.96)	0.04
HDL (1 mg/dL)			−0.44 (−0.86 to −0.02)	0.04
eGFR MDRD_186_ (1 mL/min/1.73 m^2^)			−0.46 (−0.64 to −0.28)	<0.001

**Table:** (b) LVEF model

Variables (units of increase)	Model 1 (unadjusted)		Model 2	
	*b*	*P* value	*b*	*P* value
PON1 (Q192R)	−2.96 (−4.62 to −1.29)	0.001	−2.84 (−4.54 to −1.15)	0.001
HDL (1 mg/dL)			0.07 (−0.01 to 0.15)	0.08
eGFR MDRD_186_ (1 mL/min/1.73 m^2^)			−0.02 (−0.05 to 0.01)	0.23

Data are expressed as estimate of fixed effects (*b*), 95% confidence interval, and *P* value.

## References

[1] Devereux R. B., Okin P. M., Roman M. J. (1999). Left ventricular hypertrophy as a surrogate end-point in hypertension. *Clinical and Experimental Hypertension*.

[2] Mancini G. B. J., Dahlöf B., Díez J., Cohn J. N. (2004). Surrogate markers for cardiovascular disease: structural markers. *Circulation*.

[3] Wang T. J., Evans J. C., Benjamin E. J., Levy D., LeRoy E. C., Vasan R. S. (2003). Natural history of asymptomatic left ventricular systolic dysfunction in the community. *Circulation*.

[4] Drüeke T. B., Locatelli F., Clyne N. (2006). Normalization of hemoglobin level in patients with chronic kidney disease and anemia. *The New England Journal of Medicine*.

[5] Chertow G. M., Levin N. W., Beck G. J. (2010). In-center hemodialysis six times per week versus three times per week. *The New England Journal of Medicine*.

[6] Cice G., Ferrara L., Di Benedetto A. (2001). Dilated cardiomyopathy in dialysis patients—beneficial effects of carvedilol: a double-blind, placebo-controlled trial. *Journal of the American College of Cardiology*.

[7] Park M., Hsu C.-Y., Li Y. (2012). Associations between kidney function and subclinical cardiac abnormalities in CKD. *Journal of the American Society of Nephrology*.

[8] Bansal N., Keane M., Delafontaine P. (2013). A longitudinal study of left ventricular function and structure from CKD to ESRD: the CRIC study. *Clinical Journal of the American Society of Nephrology*.

[9] Foley R. N., Parfrey P. S., Harnett J. D., Kent G. M., Murray D. C., Barre P. E. (1995). The prognostic importance of left ventricular geometry in uremic cardiomyopathy. *Journal of the American Society of Nephrology*.

[10] Zoccali C., Benedetto F. A., Mallamaci F. (2001). Prognostic impact of the indexation of left ventricular mass in patients undergoing dialysis. *Journal of the American Society of Nephrology*.

[11] Zoccali C., Bolignano D., Mallamaci F., Turner N., Goldsmith D., Winearls C., Lameire N., Himmelfarb J., Remuzzi G. (2015). Left ventricular hypertrophy in chronic kidney disease. *Oxford Textbook of Nephrology*.

[12] Mizobuchi M., Nakamura H., Tokumoto M. (2010). Myocardial effects of VDR activators in renal failure. *Journal of Steroid Biochemistry and Molecular Biology*.

[13] Yang K., Wang C., Nie L. (2015). Klotho protects against indoxyl sulphate-induced myocardial hypertrophy. *Journal of the American Society of Nephrology*.

[14] Takimoto E., Kass D. A. (2007). Role of oxidative stress in cardiac hypertrophy and remodeling. *Hypertension*.

[15] Deakin S. P., James R. W. (2004). Genetic and environmental factors modulating serum concentrations and activities of the antioxidant enzyme paraoxonase-1. *Clinical Science*.

[16] Kowalska K., Socha E., Milnerowicz H. (2015). Review: the role of paraoxonase in cardiovascular diseases. *Annals of Clinical and Laboratory Science*.

[17] Billecke S., Draganov D., Counsell R. (2000). Human serum paraoxonase (PON1) isozymes Q and R hydrolyze lactones and cyclic carbonate esters. *Drug Metabolism and Disposition*.

[18] Aviram M., Rosenblat M., Bisgaier C. L., Newton R. S., Primo-Parmo S. L., La Du B. N. (1998). Paraoxonase inhibits high-density lipoprotein oxidation and preserves its functions: a possible peroxidative role for paraoxonase. *The Journal of Clinical Investigation*.

[19] Wheeler J. G., Keavney B. D., Watkins H., Collins R., Danesh J. (2004). Four paraoxonase gene polymorphisms in 11212 cases of coronary heart disease and 12786 controls: meta-analysis of 43 studies. *The Lancet*.

[20] Lawlor D. A., Day I. N. M., Gaunt T. R. (2004). The association of the PON1 Q192R polymorphism with coronary heart disease: findings from the British Women's Heart and Health cohort study and a meta-analysis. *BMC Genetics*.

[21] Pérez-Herrera N., May-Pech C., Hernández-Ochoa I. (2008). PON1Q192R polymorphism is associated with lipid profile in Mexican men with Mayan ascendancy. *Experimental and Molecular Pathology*.

[22] Vaisi-Raygani A., Ghaneialvar H., Rahimi Z. (2011). Paraoxonase Arg 192 allele is an independent risk factor for three-vessel stenosis of coronary artery disease. *Molecular Biology Reports*.

[23] Ichikawa K., Konta T., Emi M. (2009). Genetic polymorphisms of paraoxonase-1 are associated with chronic kidney disease in Japanese women. *Kidney International*.

[24] Suehiro T., Ikeda Y., Shiinoki T. (2002). Serum paraoxonase (PON1) concentration in patients undergoing hemodialysis. *Journal of Atherosclerosis and Thrombosis*.

[25] Wilson Tang W. H., Wu Y., Mann S. (2011). Diminished antioxidant activity of high-density lipoprotein-associated proteins in systolic heart failure. *Circulation: Heart Failure*.

[26] Smith G. D., Hemani G. (2014). Mendelian randomization: genetic anchorsfor causal inference in epidemiological studies. *Human Molecular Genetics*.

[27] Stel V. S., Ioannou K., Brück K. (2013). Longitudinal association of body mass index and waist circumference with left ventricular mass in hypertensive predialysis chronic kidney disease patients. *Nephrology Dialysis Transplantation*.

[28] Quinones M. A., Waggoner A. D., Reduto L. A. (1981). A new simplified and accurate method for determining ejection fraction with two-dimensional echocardiography. *Circulation*.

[29] Tucker P. S., Dalbo V. J., Han T., Kingsley M. I. (2013). Clinical and research markers of oxidative stress in chronic kidney disease. *Biomarkers*.

[30] Suttorp M. M., Siegerink B., Jager K. J., Zoccali C., Dekker F. W. (2015). Graphical presentation of confounding in directed acyclic graphs. *Nephrology Dialysis Transplantation*.

[31] Sabri A., Hughie H. H., Lucchesi P. A. (2003). Regulation of hypertrophic and apoptotic signaling pathways by reactive oxygen species in cardiac myocytes. *Antioxidants and Redox Signaling*.

[32] Takimoto E., Champion H. C., Li M. (2005). Oxidant stress from nitric oxide synthase-3 uncoupling stimulates cardiac pathologic remodeling from chronic pressure load. *The Journal of Clinical Investigation*.

[33] Himmelfarb J., Stenvinkel P., Ikizler T. A., Hakim R. M. (2002). Perspectives in renal medicine: the elephant in uremia: oxidant stress as a unifying concept of cardiovascular disease in uremia. *Kidney International*.

[34] Locatelli F., Canaud B., Eckardt K.-U., Stenvinkel P., Wanner C., Zoccali C. (2003). Oxidative stress in end-stage renal disease: an emerging treat to patient outcome. *Nephrology Dialysis Transplantation*.

[35] Gupta N., Singh S., Maturu V. N., Sharma Y. P., Gill K. D. (2011). Paraoxonase 1 (PON1) polymorphisms, haplotypes and activity in predicting CAD risk in North-West Indian Punjabis. *PLoS ONE*.

[36] Kennedy D. J., Tang W. H. W., Fan Y. (2013). Diminished antioxidant activity of high-density lipoprotein-associated proteins in chronic kidney disease. *Journal of the American Heart Association*.

